# Role of Wnt signaling pathway in joint development and cartilage degeneration

**DOI:** 10.3389/fcell.2023.1181619

**Published:** 2023-06-08

**Authors:** Xinyan Li, Yuanyuan Han, Guimiao Li, Yingze Zhang, Juan Wang, Chen Feng

**Affiliations:** ^1^ Orthopaedic Research Institution of Hebei Province, Shijiazhuang, China; ^2^ NHC Key Laboratory of Intelligent Orthopaedic Equipment, The Third Hospital of Hebei Medical University, Shijiazhuang, China; ^3^ Department of Orthopaedic Surgery, The Third Hospital of Hebei Medical University, Shijiazhuang, China; ^4^ Department of Joint Surgery, The Third Hospital of Hebei Medical University, Shijiazhuang, China; ^5^ Hebei Orthopedic Clinical Research Center, The Third Hospital of Hebei Medical University, Shijiazhuang, China

**Keywords:** Wnt, osteoarthritis, articular cartilage, chondrocyte, mechanical loading, targeted therapy

## Abstract

Osteoarthritis (OA) is a prevalent musculoskeletal disease that affects approximately 500 million people worldwide. Unfortunately, there is currently no effective treatment available to stop or delay the degenerative progression of joint disease. Wnt signaling pathways play fundamental roles in the regulation of growth, development, and homeostasis of articular cartilage. This review aims to summarize the role of Wnt pathways in joint development during embryonic stages and in cartilage maintenance throughout adult life. Specifically, we focus on aberrant mechanical loading and inflammation as major players in OA progression. Excessive mechanical load activates Wnt pathway in chondrocytes, resulting in chondrocyte apoptosis, matrix destruction and other osteoarthritis-related changes. Additionally, we discuss emerging Wnt-related modulators and present an overview of emerging treatments of OA targeting Wnt signaling. Ultimately, this review provides valuable insights towards discovering new drugs or gene therapies targeting Wnt signaling pathway for diagnosing and treating osteoarthritis and other degenerative joint diseases.

## 1 Introduction

Articular cartilage (AC) primarily consists of chondrocytes and extracellular matrix. It provides the joint with a low-friction and wear-resistant surface, enabling it to withstand various mechanical loadings during movement, such as shear stress, hydrostatic pressure, osmotic pressure, tensile force, and pressure stress. Mechanical loading has a “double-edged sword” effect on the growth and function of chondrocyte (Jiang et al., 2021). During physiological conditions, moderate mechanical loading maintains the normal morphology and catabolic-anabolic balance of articular chondrocytes. However, when exposed to acute or long-term abnormal mechanical loading, articular cartilage degenerates because it lacks the ability to regenerate, leading to osteoarthritis (OA) (Jiang et al., 2021). Various risk factors contribute to the pathogenesis of OA, including genetics, age, obesity, abnormal mechanical overloading, and previous joint damage (Hu et al., 2018; Hunt et al., 2020). Studies have demonstrated that Wnt signaling pathway plays an important role in cartilage development and homeostasis by regulating the growth, differentiation, and proliferation of chondrocytes (Bernhard et al., 2017). This review provides a summary of how the Wnt signaling pathway affects on chondrocytes, attempting to provide potential therapeutic clues for the prevention and treatment of aberrant mechanical load-induced osteoarthritis.

## 2 Component and structure of Wnt signaling pathways

**FIGURE 1:**
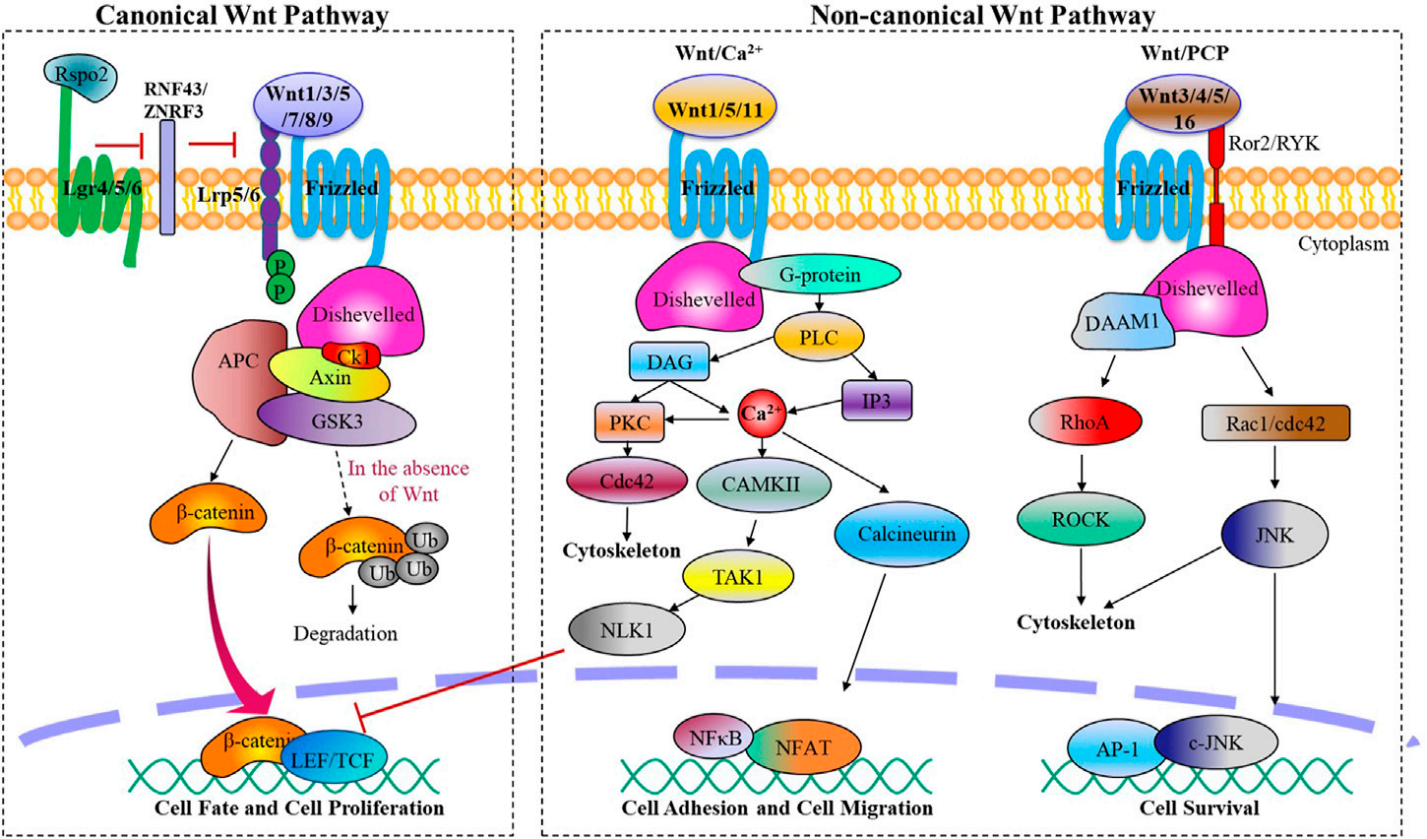
The canonical and non-canonical Wnt signaling pathways. Wnt ligands activate the canonical or non-canonical pathway depending on the receptor they bind, regulating biological processes from embryonic joint development to postnatal cartilage homeostasis.

Wnt family proteins are a type of glycoproteins that serve various functions through autocrine or paracrine secretion (Clevers and Nusse, 2012). In mammals, there exist 19 Wnt ligands that are involved in this pathway, regulating crucial biological activities during embryonic development and maintaining adult tissue homeostasis (Cho et al., 2021). Depending on different Wnt ligands and their receptors, the Wnt signaling pathway can be divided mainly two categories: β-catenin dependent canonical Wnt signaling pathway and β-catenin independent non-canonical Wnt signaling pathway. The non-canonical Wnt signaling pathway can be further subdivided into the planar cell polarity pathway (PCP) and Wnt/Ca^2+^ pathway (Figure 1).

Canonical Wnt signaling is initiated by canonical Wnt ligands, including Wnt1 Wnt3a, Wnt5a, Wnt7, Wnt8a, Wnt8b, Wnt9a, which are mediated by β-catenin. Upon binding to frizzled protein receptor (Fzd) and low-density lipoprotein receptor-related receptor 5/6 (LRP5/6), activated Fzd acts on the cytoplasmic scatter protein Dishevelled (Dsh/Dvl), destabilizing the APC/Axin/GSK-3β destruction complex and reducing its phosphorylation level of β-catenin. This process leads to an accumulation of free β-catenin in the cytoplasm, which then translocates into the nucleus and binds to TCF/LEF family members to regulate target gene expression of the Wnt signaling pathway.

Non-canonical Wnt signaling pathways, which encompass the Wnt/PCP and Wnt/Ca^2+^ pathways, are initiated by a variety of Wnt ligands such as Wnt1, Wnt3a, Wnt4, Wnt5, and Wnt11, and Wnt16. These pathways play crucial roles in processes such as cytoskeletal reorganization, chondrocyte stacking, and various phenotypic responses (Chen et al., 2021). Wnt/PCP signaling pathway is activated when Wnt ligands bind to transmembrane co-receptor tyrosine kinase-like orphan receptor 2 (Ror2) or related receptor tyrosine kinase (RYK), followed by the recruitment of scattered associated morphogenetic activator 1 (DAMM1) upon Fzd binding to Dsh. This leads to the activation of small G protein Rho or Rac1, with Rho-associated protein kinase (ROCK) activation leading to cell migration, tissue grading, and cytoskeletal changes, while Rac1 activates c-Jun N-terminal kinase (JNK), followed by activation of transcription factors c-Jun and AP-1 to promote the expression of related genes. On the other hand, Wnt/Ca^2+^ signaling results from the recruitment of Dsh to Fzd, then trimeric G protein Gα/Gβ/Gγ is recruited, subsequently activating phospholipase C (PLC). PLC then catalyzes the production of inositol triphosphate (IP3) and diacylglycerol (DAG) from phosphatidylinositol 4,5-bisphosphate in the cell membrane. IP3 releases stored intracellular calcium, resulting in the activation of calcium/calmodulin-dependent protein kinase II (CamKII) and NFAT, a transcription factor that controls cell adhesion and cell migration, as well as protein kinase C (PKC). DAG also activates PKC and mitogen-activated protein kinase (MAPK), which are involved in controlling diverse cellular activities.

## 3 The role of Wnt signaling in joint formation and cartilage homeostasis

Accumulative studies have provided substantial evidence supporting the crucial role of Wnt signaling pathways in joint development and cartilage homeostasis (Guo et al., 2004; Kahn et al., 2009; Kan and Tabin, 2013; Ray et al., 2015; Chen et al., 2017) (Table 1). Specifically, the canonical Wnt/β-catenin signaling pathway plays a significant role in regulating chondrocyte phenotype, maturation, and function during the cartilage development process, which is crucial for cartilage defining cartilage boundaries and endochondral ossification (Tamamura et al., 2005). Furthermore, Wnt signaling interacts with other signaling pathways, such as TGF-β, Bmp/Smad, Ihh, to form a complex network of cell signaling pathways which collectively regulate chondrocyte growth and development.

**TABLE 1:** Role of Wnt ligands in the formation of joints and the pathogenesis of osteoarthritis.

Wnt ligand	Effect on joints	Model	References
Wnt1	Upregulates matrix-degrading enzymes such as ADAMT5, ADAMT7 and MMP13	Human	Fang et al. (2021)
Wnt3a	Reduces ECM-related proteins and/or promotes ECM catabolic enzyme secretion	Mouse, Rat, Human	Liu et al. (2016), Thomas et al. (2021), Xie et al. (2023)
	Promotes chondrocyte hypertrophy	Mouse, Human	Wang et al. (2019), Bertrand et al. (2020)
	Maintains the balance of chondrocyte differentiation and dedifferentiation by Ca^2+^/CaMKII-dependent and β-catenin-dependent pathways reciprocally inhibitory	Human	Nalesso et al. (2011)
Wnt4	Maintains joint cell identity and regulates chondrocyte maturation during embryonic development; regulates chondrocyte maturation at the transition from pre-hypertrophic to hypertrophic chondrocytes	Mouse	Später et al. (2006)
	Conditional loss of Wnt4 in the limb mesenchyme are also more prone to develop spontaneously OA-like joint alterations with age	Mouse	Teufel et al. (2022)
Wnt5a	Inhibits type II collagen expression	Rabbit	Ryu and Chun (2006)
	Upregulates MMP1, MMP13 and inhibits of aggrecan expression	Human	Huang et al. (2017)
	A potential main mediator to modulate chondrocyte dedifferentiation and re-differentiation	Human	Kutaish et al. (2022)
Wnt5b	Promotes the chondrocyte hypertrophy, proliferation, and differentiation	Mouse	Yang et al. (2003)
	Highly expressed in dedifferentiated chondrocytes	Human	Kutaish et al. (2022)
Wnt7a	Causes dedifferentiation of chondrocytes	Rabbit	Ryu and Chun (2006)
	Inhibits the progressive increase of joint matrix metalloproteinases (MMPs) activity in DMM animals, thereby promoting the integrity of articular cartilage	Mouse, Human	Gibson et al. (2017)
Wnt7b	Modulates chondrocyte dedifferentiation and re-differentiation	Human	Kutaish et al. (2022)
Wnt8a	Shows higher expression in patients with osteoarthritis, in comparison with those with hip fractures	Human	García-Ibarbia et al. (2013)
Wnt9a	Transforms chondrocytes into fibroblast-like cells producing a different type of collagen	Chicken	Später et al. (2006)
	Regulates the expression of Indian hedgehog, thereby influencing the onset of hypertrophy	Mouse	Später et al. (2006)
	Conditional loss of Wnt4 in the limb mesenchyme is prone to develop spontaneously OA-like joint alterations with age	Mouse	Teufel et al. (2022)
Wnt10a	Promotes the expression of inflammation cytokines in OA patients SMDCs; shows a protective effect on cartilage integrity in rat model	Human, Rat	Huang et al. (2020)
Wnt10b	The expression of wnt10b is parallel to the severity of inflammation cell infiltration and tissue fibrosis in RA and OA synovium	Human	Imai et al. (2006)
	Promotes chondrocyte proliferation in the proliferating chondrocyte layer	Mouse	Matsuura et al. (2020)
Wnt11	Stimulates type II collagen expression	Rabbit	Ryu and Chun (2006)
Wnt16	Maintains a balanced canonical WNT signaling and prevents detrimental excessive activation; supports the lubrication expression of progenitor cells in the superficial zone of cartilage	Mouse	Nalesso et al. (2017)
	Activates PCP/JNK and interacts with mTORC1-PTHrP pathway to inhibit chondrocyte hypertrophy	Mouse	Tong et al. (2019)

The Wnt signaling pathway is essential for joint initiation. On one hand, Wnts can inhibit chondrogenesis by downregulating the expression of Sox9 and Noggin, which promotes the de-differentiation of chondrocyte into interzone cells (Hartmann and Tabin, 2001). On the other hand, there is an overlapping expression pattern of Wnt4, Wnt9a, and Wnt16 in the joint region, where they can induce interzone marker gene expression, such as Gdf5, CD44, Chordin, and autotaxin (Guo et al., 2004). The expression of exogenous Wnt9a through retrovirus at the joint formation site can cause the failure of the joint development in that location. This implies that Wnt9a, which is regulated by transcription factor c-Jun (Kan and Tabin, 2013), can act as a joint formation inhibitor to ensure that newly-formed joints only developed at specific distances away from the previously-formed joints. Overexpression of Wnt9a can inhibit the chondrogenesis properties of cells, while ectopic expression of Wnt9a leads to aberrant expression of a series of joint marker genes. However, Wnt9a is not an indispensable factor for joint formation, because the absence of Wnt9a only leads to synovial chondroid metaplasia in certain joints, and does not affect the formation of joints (Später et al., 2006). Singh and others suggested that interplay between Wnt and BMP signaling is crucial in defining the emerging joint territories (Singh et al., 2018).

During joint development, the Wnt signaling pathways crosstalk with other pathways, such as the TGF-β superfamily and Ihh signaling pathway to determine chondrocyte fate and maintain joint homeostasis maintenance (Seo and Serra, 2007; van den Bosch et al., 2016). The growth plate chondrocyte and articular chondrocyte originate from the same population. The type of cartilage that the cells will develop into is determined by differential exposure of the cartilage anlagen to BMP or Wnt signaling (Ray et al., 2015). TGF-β activates β-catenin signaling through the interaction between SMAD3 and β-catenin. The protein complex, SMAD3/4/β-catenin contributes to the nuclear translocation process of β-catenin. In addition, Wnt3a mediated canonical Wnt signaling decreases the levels of phosphorylation signaling of SMAD2 and SMAD3, and increases WISP1, SMAD 1, SMAD5, and SMAD8 in chondrocytes (Oka et al., 2007; Chen et al., 2017; Singh et al., 2018; Zhou et al., 2018; Cherifi et al., 2021). Moreover, the canonical Wnt/β-catenin signaling pathway and Ihh signaling pathway collaborate to regulate the formation of synovial joints. Wnt9a has the potential to influence the differentiation of pre-hypertrophic chondrocytes by regulating the expression of Ihh signaling (Später et al., 2006).

In a study of Wnt signaling using transgenic mice carrying the TOPgal reporter gene, a small number of TOPgal positive cells were found in the articular cartilage of adult mice. This finding suggests that Wnt signaling maintains a stem cell/progenitor cell bank in adult life to facilitate the regeneration and repair of articular cartilage (Yamagami et al., 2009).

## 4 The role of Wnt signaling in cartilage degeneration

Among various factors leading to cartilage degeneration, abnormal mechanical stimulation plays a major role. Under physiological conditions, mechanomechanical signals from chondrocytes usually promote chondrocyte proliferation, enhance chondrocyte extracellular matrix (ECM) anabolism, inhibit inflammatory responses, and maintain cartilage integrity (Grodzinsky et al., 2000). For example, physiological tensile strain (7.5%, 1HZ for 30min) could induce the transcription of Col2a1, Aggrecan and SOX9 (Thomas et al., 2011). However, excessive mechanical loading increases Wnt signaling in articular cartilage, which triggers osteoblastogenesis and metalloproteinase production. These processes destroy articular chondrocyte properties, differentiate them into hypertrophic chondrocytes, eventually causing necrosis or apoptosis. Consequently, the major component of articular cartilage ECM proteins shift from type II to type X collagen, and remodeling of articular cartilage occurs. On the contrary, reduced Wnt signaling stimulates chondrogenesis (Torzilli et al., 1999; Haapala et al., 2000; Deshmukh et al., 2018).

Studies on OA patients, surgery-induced OA mouse models, aged mice, and spontaneous guinea pig osteoarthritis model reveal that there is an upregulation of β-catenin expression along with the upregulation of MMPs and ECM degradation, which are indicative of chondrocyte hypertrophic differentiation (Yuasa et al., 2008; Ma et al., 2019; Ding et al., 2020). After mechanical injury of articular cartilage in adults, the Wnt inhibitor frizzled-related protein (FRZB) was continuously downregulated within 24 h. Moreover, a significant upregulation of axial protein 2 and c-JUN expression was detected (Dell’Accio et al., 2006). Fang et al. discovered that excessive mechanical load could induce abnormal activation of Wnt1 and Wnt3a signals, resulting in a significant increase in matrix-degrading enzymes such as ADAMT5, ADAMT7 and MMP13 (Fang et al., 2021). Furthemore, increased ECM deposition leads to the decline of β-catenin levels, which determines the response of chondrocytes to mechanical loading (Praxenthaler et al., 2018). Thus, in order to enhance proteoglycan synthesis, it is crucial to maintain low levels of Wnt activity in cartilage tissue that undergoes excessive mechanical loading as it enables chondrocytes to mount anabolic responses and enhance proteoglycan synthesis.

The canonical Wnt/β-catenin pathway plays a significant role in regulating cartilage chondrocyte mechanotransduction. Wnt3a is a critical mediator of the canonical Wnt signaling pathway. Following treatment with recombinant Wnt3a for 24h, there was a marked decrease in the expression of Aggrecan and SOX9, and partial transport of β-catenin to the nuclear. In the mechanical transduction mediated by Wnt3a, β-catenin was widely distributed in both the nucleus and cytoplasm. Furthermore, catabolic genes such as MMP3 and ADAMTS-4 were upregulated in Wnt3a-stimulated chondrocytes (Thomas et al., 2011). In addition, the study of Wnt3a by Nalesso et al. showed that Wnt3a simultaneously activates both the Wnt/β-catenin and Wnt/Ca^2+^ pathways in a dose-dependent manner, and promotes chondrocyte proliferation and differentiation through the Wnt/β-catenin and Wnt/Ca^2+^ signaling pathways, respectively. These two pathways inhibit each other. After treating chondrocytes with the canonical Wnt signaling pathway inhibitor DKK1, they found that blocking the Wnt/β-catenin pathway would also lead to the dedifferentiation of articular chondrocytes by inhibiting the Wnt/Ca^2+^ pathway (Nalesso et al., 2011).

Wnt5a is a representative Wnt protein found in human articular cartilage with OA changes (Kumawat and Gosens, 2016). It activates several proteins such as CaMKII, JNK, p38, ERK1/2, p65 and Akt, promoting the non-canonical Wnt signaling pathway. Like Wnt3a, Wnt5a also promotes chondrocyte catabolic signaling, upregulating MMP1 and MMP13 while inhibiting aggrecan expression (Huang et al., 2017). In addition, Wnt5a-mediated Wnt signaling could also affect the expression of inflammatory factors. Lambert C et al. found that Wnt5a and LRP5 were upregulated, while FZD2 and Dkk-3 were downregulated in patients with knee osteoarthritis through gene expression profiling. Furthermore, at the protein level, Wnt5a was confirmed to be the most upregulated gene in this signaling pathway (Lambert et al., 2014). Sun et al. studied the effect of Secreted frizzled-related protein 5 (SFRP5), an endogenous inhibitor of Wnt5a, on the inflammatory response of OA and found that overexpression of SFRP5 reduced the expression of inflammatory factors such as IL-1β, IL-6, TNF-α and ROS. Additionally, they demonstrated that SFRP5 attenuates LPS-induced OA inflammation and chondrocyte apoptosis through the Wnt5a/JNK pathway (Sun et al., 2021).

Unlikely Wnt3a and Wnt5a, some other Wnt ligands could play beneficial role for maintaining the integrity of articular cartilage. For example, intra-articular administration of lentivirus Wnt7a in destabilized medial meniscus (DMM) mice could significantly reduce articular cartilage injury. Through inhibiting the progressive increase of joint matrix metalloproteinases (MMPs) activity in DMM animals (Gibson et al., 2017). Also, based on another report, the signaling activity of Wnt/β-catenin is highly specific in the superficial zone (SFZ) of adult mice articular cartilage, where a high expression of proteoglycan 4 (Prg4) is indicated. In SFZ-specific β-catenin stabilized mice, Prg4 expression was enhanced, leading to the inhibition of articular cartilage degeneration. Moreover, following mechanical loading, Wnt5a, Wnt5b, and Wnt9a were upregulated in SFZ, contributing significantly to Prg4 transcription. In addition, mechanical loading and activation of the Wnt/β-catenin signaling pathway increase mRNA levels of Creb1, an effective transcription factor of Prg4, thereby playing an important role in maintaining articular cartilage homeostasis (Xuan et al., 2019).

Wnt16 plays a crucial role in regulating the relationship between canonical and non-canonical Wnt signaling. It has been demonstrated that Wnt16 supports the phenotype and lubrication expression of progenitor cells in the superficial zone of cartilage, and Wnt16 knockout mice show more severe osteoarthritic phenotype after DMM surgery, due to reduced lubrication expression and increased chondrocyte apoptosis (Nalesso et al., 2017). Although not expressed in articular cartilage of healthy adult mice, significant upregulation of Wnt16 and β-catenin localization is observed at mRNA and protein levels after cartilage injury in both *in vivo* and *in vitro* models (Dell’Accio et al., 2008). Wnt16 could inhibit chondrocyte hypertrophy during the development of OA through the planar cell polarity (PCP)/JNK-mTORC1-PTHrP cascade, thereby inhibiting cartilage catabolism (Tong et al., 2019). In addition to activating the Wnt/PCP pathway, Wnt16 balances canonical Wnt signaling. *In vitro*, high doses of Wnt16 weakly activated the canonical Wnt signaling pathway, but in co-stimulation experiments, Wnt16 reduced the ability of Wnt3a to activate the canonical Wnt pathway, prevented its overactivation, and maintains cellular homeostasis while preventing chondrocyte apoptosis (Nalesso et al., 2017). Finally, the Wnt/planar cell polarity pathway synergizes with activated receptor tyrosine kinase-like orphan receptor 2 (ROR2), resulting in intracellular phosphorylation of Vangl2 and ultimately inhibition of the canonical Wnt signaling path way (Thorup et al., 2020). At the molecular level, different types of Wnt signaling regulate each other, maintain relative balance, and jointly contribute to the maintenance of cartilage homeostasis.

In addition to Wnt proteins themselves, regulatory proteins involved in the Wnt/β-catenin signaling pathway have been found to play vital roles in the progression of osteoarthritis. R-spondin proteins are secreted extracellular ligands that could activate Wnt/β-catenin signaling, and could be regulated by this pathway (Friedman et al., 2009; de Lau et al., 2011). Initially, it was believed that R-Spondins positively regulate Wnt signaling by directly interacting with Frizzled-8 and Lrp5/6, while competing with Dkk1 (Binnerts et al., 2007; Kim et al., 2008). However, later evidence revealed that this effect relies on leucine-rich repeat-containing G-protein-coupled receptor 5 (Lgr5), a member of G protein-coupled receptor family (Carmon et al., 2011). Kinji Ohno and others reported that Rspo2 promotes chondrocyte differentiation from proliferating chondrocytes into hypertrophic chondrocytes during endochondral ossification through activating Wnt/β-catenin signaling (Takegami et al., 2016). Recent reports suggest that Rspo2-induced activation of Wnt signaling plays a role in OA progression. Reduced expression of Rspo2 in osteoblasts is responsible for the downregulation of Wnt signaling and abnormal mineralization in OA patients (Abed et al., 2011). In contrast, Rspo2 is in a high level of protein expression in the synovial fluid of OA patients (Okura et al., 2019), which partially contributes to synovial macrophage M1 polarization (Zhang et al., 2018). Most recently, Rspo2 was found to be the mediator in charge of cross-talking between synovial fibroblasts, macrophages, and chondrocytes, contributing to OA symptoms in multiple tissues after joint trauma (Knights et al., 2023).

Lgr5, an important Wnt signaling target gene, has recently been dicovered to express in the articular zone of the temporomandibular joint in mice and the deep zone of articular cartilage in OA patients (Zhou et al., 2018; Lee et al., 2020). Studies on joint development of embryonic mice have indicated that Lgr5-expressing joint progenitor cell populations play an important role in the formation of joint tissues, including the cruciate ligament, synovium, and articular cartilage. Lgr5^+^ cells can promote the repair of joint defects by rebuilding the articular cartilage (Feng et al., 2019). Through binding to Lgr5, Rspo2 can displace Wnt antagonists such as membrane-associated ring finger protein 43 (Rnf43) zinc and ring finger 3 (Znrf3) from Lrp5/6, thereby activating Wnt signaling (Zhou et al., 2018). This binding could be disrupted by Mianserin, a tetracyclic antidepressant, in chondrocytes, thereby reducing the effect of Rspo2-induced activation of Wnt/β-catenin. In the articular chondrocytes of rat OA model, intraarticular administration of Mianserin could suppress abnormally activated Wnt/β-catenin signaling and play a protective role against OA (Okura et al., 2019). Together, these findings suggest that RSPO2-induced, LGR5-dependent Wnt signaling-positive feedback loop exerts an important impact on formation and homeostasis of articular cartilage, and can be a potential therapeutic target for OA treatment. However, further studies are required to provide direct evidence with respect to the role of Lgr5 in articular cartilage degeneration in OA.

Furthermore, in the regulation of cartilage metabolism, Wnt signaling could cooperate with mechanical force-mediated calcium signaling pathway. Mechanosensitive ion channels Piezo1/2 and transient receptor potential vanilloid 4 (TRPV4) are two key calcium channels that respond to mechanical forces, and recent studies have shown that the Wnt signaling pathway was involved in their mediated regulation of bone metabolism. Piezo1 channel responds to hydrostatic pressure (HP) and regulates Wnt expression (Wnt5b and wnt16) and ciliogenesis to promote odontoblast differentiation. Also, the Peizo1 agonist Yoda1 increased Wnt16 expression (Miyazaki et al., 2019). The stimulation of Piezo1 by fluid shear stress induced the expression of Wnt1 in part via YAP1 and TAZ, thus promoted bone anabolism (Li et al., 2019). *In vitro* and *in vivo* findings showed that Piezo1/2 deletion could reduce osteogenic differentiation of bone mesenchymal stem cells (MSCs) through Wnt/β-catenin and Yap1 pathways. In contrast, activation of Piezo1/2 induced osteogenic differentiation by activating Wnt and YAP signaling pathway (Zhou et al., 2020). In addition, Piezo1 and YAP/TAZ may also act as downstream effectors of Wnt5a in MSCs (Tao et al., 2019). The activation of TRPV4 could promote the nuclear translocation of NFATc1, activate the Wnt/β-catenin signaling pathway, and induce osteogenesis (Hou et al., 2019). However, the interaction between Piezo1 or TRPV4 channels and the Wnt pathway has been less studied in OA, therefore, given the important role of mechanoreceptor Piezo1 and TRPV4 channels in OA, more research may be needed.

In conclusion, Wnt signaling plays an important role in regulating the fate and metabolism of cartilage chodrocytes in turns of mechanotransduction and inflammation. The impact of Wnt signaling on OA is intricate since loss and gain of function of the Wnt/β-catenin signaling pathway in chondrocytes both could potentially leading to joint damage or exacerbate osteoarthritis. While strong inhibition of active Wnt/β-catenin signaling in cartilage leads to complete cartilage destruction and cell death in 15-month-old mice with OA, loss of function of the Wnt inhibitor is prone to increase susceptibility to OA in both mice and humans (Nalesso et al., 2017; Cherifi et al., 2021).

## 5 Wnt signaling modulators and potential targeted OA therapies

According to the Chinese Guidelines for the Diagnosis and Treatment of Osteoarthritis (2021 Edition), the current clinical treatment of OA is still based on relieving pain and inflammatory symptoms as well as joint replacement surgery, there is no effective treatment that can prevent or delay degenerative changes in the joint, and it is urgent to develop more targeted treatments that act on specific signal transduction pathway. At present, treatment of osteoarthritis remains largely limited to steroidal or nonsteroidal anti-inflammatory drugs that only relieve symptoms of pain and inflammation (Latourte et al., 2020). With the deepening of understanding and research on articular cartilage, new therapies can be achieved by targeting the Wnt signaling pathway to promote cartilage repair or limit bone remodeling and improve prognosis.

Different inhibitors can act on different targets of Wnt signaling pathway and regulate anabolism and catabolism of chondrocytes, to achieve the purpose of improving OA (Table 2). On the one hand, some inhibitors act on extracellular Wnt signals and FZD and other targets. SFRP3 is a soluble antagonist of the Wnt signaling pathway, and single nucleotide polymorphisms (SNP) in SFRP3 have been reported to be associated with an increased risk of OA in weight-bearing regions of the joint (Loughlin et al., 2004; Zhou et al., 2017). DKK1 is a negative regulator of the canonical Wnt signaling pathway (Fulciniti et al., 2009). Transgenic expression of DKK1 in chondrocytes has been reported to attenuate surgery-induced cartilage destruction in OA knees (Huang et al., 2018). Another potent Wnt signaling antagonist is sclerostin, which is increased in surgically induced OA mouse, sheep knee OA cartilage, and human knee OA cartilage. Treatment of ovine cartilage explants with sclerostin inhibited the Wnt/β-catenin signaling pathway, mainly by reducing Mmp, Adamts, Acan, and Col2a1 gene expression to produce anti-catabolic effects (Chan et al., 2011). Fibulin-4 is an ECM protein involved in the development of connective tissue and the formation of elastic fibers. Lei et al. investigated the function of Fibulin-4 in cartilage development and found that Fibulin-4 regulates Wnt/β-catenin signaling by promoting the expression of Wnt-3a and β-catenin and reducing the activity of GSK-3β in chondrocytes of human osteoarthritis, thus affecting chondrocyte differentiation, while Fibulin-4 effectively inhibits the Wnt inhibitor DKK 1 and plays an indirect role in regulating the Wnt signaling pathway (Shangguan et al., 2017).

**TABLE 2:** Wnt related pharmaceutical molecules treating for osteoarthritis.

Modulation	Modulator	Effect on Wnt signaling	Function in OA	References
Extracellular Modulation	FRZB (sFRP-3)	Soluble Wnt Inhibitors	Inhibits the activation of the Wnt/β-catenin canonical signaling pathway by binding to Wnt3a and thus reduced cartilage damage in OA mice	Ding et al. (2020)
	Wnt16	Weak activators	Regulates chondrocyte differentiation through the Wnt/PCP pathway	Tong et al. (2019)
	DKK1	Inhibitors of FZD/LRP interaction	Inhibits the activation of Wnt/β-catenin canonical signaling pathway by binding to Wnt3a and inhibits the expression of MMP13 and Adamts4 and the destruction of OA cartilage	Oh et al. (2012)
	Sclerostin (SOST)		Inhibits Wnt/β-catenin signaling and catabolic metalloproteinase (MMP and ADAMTS) expression, decreased mRNA levels of aggrecan, collagen II and tissue inhibitors of metalloproteinaes (TIMPs)	Chan et al. (2011)
	Fibulin-4		Activates Wnt/β- catenin signaling by inhibiting the Wnt inhibitor DKK1, and attenuated the production of ECM (Col2a1, Col10a1 and Acan) and the expression of chondrocyte differentiation (Runx2, Sox2, and Sox9)	Shangguan et al. (2017)
Intracellular Modulation	XAV-939	Tankyrase inhibitor	Promotes anti-catabolism against chondrocytes by inhibiting the Wnt/β-catenin canonical signaling pathway	Lietman et al. (2018)
	GIN	Inhibitors of GSK-3β	Downregulates the transduction of canonical Wnt signals by promoting the degradation of β-catenin, maintained the chondrocyte phenotype and participated in the maintenance of the chondrocyte extracellular matrix	Miclea et al. (2011)
	ICAT	Inhibitors of TCF	Negatively regulates Wnt signaling by inhibiting the interaction between β -catenin and TCF-4	Tago et al. (2000)
	SAH-Bcl9 StAx-35R	Inhibitors of TCF/LEF promoter	Upregulates SOX9 and aggrecan (ACAN) expression and reduced COL10A1 gene expression by inhibiting the TCF/LEF promoter activity of the Wnt3a-induced Wnt/β-catenin canonical signaling pathway	Held et al. (2018)
	DOT1L	Epigenetic modulators	Reduces the expression of β-catenin-related transcription factors LEF1 and TCF1, prevented the over-activation of Wnt by negatively regulating SIRT1, and thereby maintained cartilage homeostasis	Monteagudo et al. (2017)
Pathway activity modulation	Lorecivivint (SM04690)	Indirectly interact with Wnt components	Affects the activity of Wnt pathway and reduced the release of chondrocyte matrix degrading enzyme by inhibiting CLK2 and DYRK1A, and Phase III clinical trials are currently under way	Yazici et al. (2020), De Palma and Nalesso (2021)

On the other hand, some inhibitors inhibit the transduction of Wnt signaling pathway by targeting pathway sites in the cytoplasm, or act to block the transcriptional activity of TCF/LEF in the nucleus. Intra-articular injection of GIN, a GSK-3 inhibitor, induced cartilage surface fibrillation and reduced glycosaminoglycan expression and chondrocyte dysfunction by downregulating the canonical Wnt signaling pathway (Miclea et al., 2011). Li et al. investigated the function of N-cadherin peptidomimetic peptide and found that N-cadherin peptide could increase the expression of GSK-3β, decrease the nuclear localization of β-catenin, and inhibit the transcriptional activity of β-catenin/LEF-1/TCF complex, thereby inhibiting the transduction of classical Wnt signaling pathway and enhancing the chondrogenic differentiation of human MSCs (Li et al., 2017).

Furthermore, blockade of canonical Wnt signaling by two small molecule inhibitors, SAH-Bcl9 and StAx-35R, suppressed chondrocyte phenotypic transfer in preserved cartilage at early stages of OA, resulting in increased SOX9 and ACAN and decreased COL10A1 gene expression (Held et al., 2018). DOT1L, an enzyme participating in the histone methylation, is reported to negatively regulate Wnt signaling pathway through inhibition of SIRT1, and thus play roles in protect against osteoarthritis (Monteagudo et al., 2017). Cyclin D1, an indispensable factor for regulating the cell cycle, is reported to be an important molecule involved in the pathological process of osteoarthritis (Zhu et al., 2016). The authors’ team further clarified that cyclin D1 regulates chondrocyte proliferation and apoptosis in OA by mediating Wnt3/β-catenin signaling (Chen et al., 2019).

In addition, inhibitor have been entered into Phase III clinical trials (De Palma and Nalesso, 2021). SM04690 is a small-molecule Wnt signaling inhibitor developed by cell high-throughput screening (Deshmukh et al., 2018), which is used as a local intra-articular (IA) injection for knee OA. *In vitro*, studies have shown that SM04690 can effectively inhibit the Wnt pathway, and Wnt gene expression is significantly downregulated 48 h after treatment of human MSCs. After 21 days, SM04690 induces downregulation of osteogenic gene expression and upregulation of cartilage gene expression (Yazici et al., 2017). In addition, SM04690 was found to effectively attenuate overload induced cartilage injury by inhibiting Wnt signaling pathway through up-regulating Wnt inhibitory gene, decreasing β-catenin expression and nuclear localization in a rat knee osteoarthritis model (Deshmukh et al., 2018). Next, to further clarify the molecular mechanism and downstream pathway of SM04690, the authors’ team isolated and cultured primary chondrocytes for *in vitro* studies and found that SM04690 inhibited the canonical Wnt/β-catenin pathway, upregulated the expression level of Wnt16, increased the expression of cartilage anabolic factors COL2A1, SOX9, and aggrecan, while inhibiting the expression of cartilage catabolic factor MMP13, thus playing a role in protecting chondrocytes (Hua et al., 2022).

Currently, it has been shown that clinical drugs applied in other fields can also protect cartilage and improve OA progression through specific Wnt signaling pathways. Okura et al. found that the content of R-spondin 2 in synovial fluid of patients with osteoarthritis gradually increased with the severity of OA, and mianserin could prevent and improve articular cartilage degradation in a mouse model of knee OA with medial meniscus instability by inhibiting R-spondin 2-induced Wnt/β-catenin signaling pathway in chondrocytes (Okura et al., 2019). Strontium ranelate (SrR) is an antiosteoporosis drug that has been proven to affect OA by inhibiting the Wnt/β-catenin signaling pathway, promoting BMSCs chondrogenic differentiation and accelerating cartilage regeneration (Yu et al., 2021). Salinomycin, an anti-tumor drug, slows OA severity by inhibiting Lrp6 phosphorylation, which in turn decreases activation of the Wnt/beta-catenin signaling pathway (Chen et al., 2022).

At present, it has been shown that the active ingredients of Chinese herbs also have positive effects on slowing OA progression. Achyranthes bidentata is a commonly used traditional Chinese medicine for the treatment of osteoarthritis, of which Achyranthes bidentata saponin D (Ach-D) is the main therapeutic component. By evaluating treatment after anterior cruciate ligament transection combined with medial meniscectomy in rats, Xie et al. found that Achyranthes saponin targets Wnt3a, inhibits the Wnt signaling pathway, reduces inflammation and cartilage lesions, thereby playing a role in slowing OA (Xie et al., 2023). Huang et al. used the Hulth method to construct a rat knee OA model and analyzed isolated and cultured chondrocytes. The results showed that tetramethoxyflavone (TMF), the main component of Murraya exotica L., showed chondroprotective activity by up-regulating Foxo3a expression and inhibiting miR-29a/Wnt/β-catenin signaling pathway activity (Huang et al., 2019). Polydatin is a natural active substance extracted from Polygonum cuspidatum and Fallopia multiflora. Several studies have shown that PD has anti-inflammatory, antioxidant, and anti-tumor activities, and has a positive effect on osteogenic differentiation and inflammatory response in OA. The authors established a rat OA model by surgical ACLT and injected PD intra-articularly, and showed that PD significantly reduced articular cartilage degeneration and protected cartilage degradation by regulating the Wnt/β-catenin signaling pathway (Hu et al., 2022). Palmatine, a structural analog of berberine, is useful in the treatment of OA by the inhibition of the Wnt and Hedgehog signaling pathways (Zhou et al., 2016). Scutellarin has many beneficial effects such as anti-inflammatory, antioxidant and anticoagulant effects, it has been applied to a variety of diseases. However, it has been reported that scutellarin can downregulate the expression levels of Wnt3a, Frizzled7, MMP1, MMP13 and ADAMTS-5, promote the expression of collagen II and aggrecan, and inhibit the migration of β-catenin, thereby significantly inhibiting cartilage degradation in DMM-induced OA mice (Liu et al., 2020).

In addition, dietary nutrient intake in daily life can also have an impact on the development of osteoarthritis. Several studies have now shown that some dietary micronutrient supplementation may protect against OA (Thomas et al., 2018), which is beneficial in improving symptoms and improving clinical treatment outcomes. Bai et al., in a DMM-induced OA model, found that low magnesium conditions inhibited chondrocyte autophagy by activating the Wnt/β-catenin signaling pathway, thereby decreasing the expression of anabolic factors, and increasing the expression of catabolic factors in chondrocytes by controlling the daily diet of mice (Bai et al., 2022).

Increasing evidence suggests that Wnt signaling plays an important role in maintaining joint homeostasis, particularly in chondrocytes. When joints are subjected to abnormal mechanical loading, the balance of Wnt signaling in the body is disrupted, followed by cartilage damage, leading to osteoarthritis at last. Therefore, it is necessary to further understand the role of Wnt signaling pathway in chondrocyte homeostasis, and new targeted drugs or gene therapy targets can also be developed through this signaling pathway to provide a new direction for the diagnosis and treatment of mechanical load-mediated osteoarthritis and other diseases.

## 6 MicroRNAs regulating Wnt signaling pathway in osteoarthritis

When exposed to circulating hydrostatic pressure (HP), the expression levels of miR-27a/b, miR-146a/b, miR-140, and miR-365 and their target genes (MMP-13, ADAMTS-5, HDAC-4) were regulated in OA chondrocytes, by activating the Wnt/β-catenin pathway (Cheleschi et al., 2017). Increasing evidence has shown that regulating the expression of certain microRNAs (miRs) can affect chondrocyte properties and participate in the progression of OA. These miRs were classified into three types based on their targets in the Wnt signaling pathway.

The first type includes miRs that directly act on Wnt signals, such as miR-154-5p, miR-92-3p, miR-497-5P, miR-26b, miR-410, miR-203 and miR-146a. Some miRs act on DKK-1, including miR-320a, miR-335-5p. Other miRs act on Frizzled, including miR-1 and miR-29 (Shang et al., 2021). Mao et al. found that exosomal miR-92a-3p regulated cartilage development and homeostasis by directly targeting Wnt5a, inhibiting chondrogenic differentiation and reducing cartilage matrix synthesis (Mao et al., 2018). In chondrocytes, miR-1 increases β-catenin phosphorylation by binding to FZD7, blocks downstream β-catenin signaling pathways, and ultimately weakens the expression of genes that regulate catabolic enzyme activity (Yang et al., 2021).

In addition, it has been found that the Wnt/β-catenin pathway can significantly downregulate the downstream coding products MMP13, ADAMT5, IGFBP5, and HDAC4 by regulating specific miRs associated with OA, such as miR-27a/b, miR-140, miR-146a/b, and miR-365 (Cheleschi et al., 2017).

Secondly, there are miRs that act on β-catenin and related proteins, such as miR-29c-3p for Dsh/Dvl, miR-26b for GSK-3, and miR-142-3p for APC. And miR-320c and miR-10a directly acting on β-catenin. They can directly or indirectly act on β-catenin in the cytoplasm, regulate different processes such as cell proliferation, migration, and regeneration by transducing extracellular signals, and then affect the progression of OA (Shang et al., 2021). Hu et al. showed that miR-320c could directly target β-catenin mRNA to regulate β-catenin expression, inhibit MMP-13 expression, increase COL2A1 expression, and reduce the relative transcriptional activity of β-catenin/TCF complex, inhibiting osteoarthritis chondrocyte degeneration by inhibiting the canonical Wnt signaling pathway (Hu et al., 2019). Wang et al. found that miR-200a-3p could indirectly regulate the expression level of β-catenin by binding to FoxC1, thereby upregulating the expression of ADAMTS-5, fibronectin, matrix metalloproteinase 3 (MMP3) and matrix metalloproteinase 13 (MMP13), and ultimately alleviating OA inflammation (Wang et al., 2020).

Finally, some miRNAs contribute to the transcription of TCF/LEF in the chondrocyte nucleus. Studies have shown that miR-365, miR-222, miR-140 and others directly target HDAC that inhibits TCF-4 degradation. MiR-145, miR-101, and miR-615-3P target SOX-9 to affect the nuclear phosphorylation of β-catenin, and thus regulate the transduction of Wnt signaling pathway (Shang et al., 2021). Based on a previous study that miR-138 expression inhibits cartilage tissue destruction in OA patients (Wei et al., 2017) and can maintain the chondrocyte phenotype (Seidl et al., 2016), Xu et al. finally clarified that increased miR-138 expression may lead to downregulation of NEK2 by studying chondrocytes in OA mice, Phosphorylate LEF1 positively regulates Wnt signaling pathway, thereby promoting chondrocyte proliferation and inhibiting chondrocyte apoptosis in OA through the WNT/β-catenin signaling pathway (Xu et al., 2019). MiR-140–5p via RalA enhanced the proliferation and migration of articular chondrocytes without the destruction of extracellular matrix, successfully promoted cartilage regeneration and prevented OA in a rat model (Tao et al., 2017).

## 7 Conclusion

There are two fates of chondrocytes, either undergoing endochondral ossification and being replaced by osteocytes, or becoming long-lasting articular chondrocytes residing on the joint surface. The Wnt signaling pathways play a critical role in both processes, especially in joint formation and articular cartilage homeostasis. Targeting the overactive Wnt/β-catenin signaling in OA process has emerged as a strong and promising preventive strategy against OA. Nevertheless, the modulators and networks responsible for the overactive Wnt signaling in OA are not well characterized. Although the modulators mentioned herein are not exhaustive, they can serve as clues to find new drug or gene therapies that target the Wnt signaling pathway for diagnosing and treating osteoarthritis and other degenerative joint diseases.
